# Construct validity of advanced practice nurse core competence scale: an exploratory factor analysis

**DOI:** 10.1186/s12912-023-01203-1

**Published:** 2023-03-02

**Authors:** Sek Ying Chair, Frances Kam Yuet Wong, Denise Bryant-Lukosius, Ting Liu, Krista Jokiniemi

**Affiliations:** 1 The Nethersole School of Nursing, Faculty of Medicine, The Chinese University of Hong Kong, N.T. Shatin, Hong Kong SAR, China; 2 The Hong Kong Academy of Nursing, Lai Chi Kok, Kowloon, Hong Kong SAR China; 3 School of Nursing, The Hong Kong Polytechnic University, Hung Hom, Kowloon, Hong Kong SAR China; 4 School of Nursing, McMaster University, Hamilton, ON Canada; 5 School of Nursing, Sun Yat-Sen University, Guangzhou, Guangdong China; 6 Department of Nursing Science, Faculty of Health Sciences, University of Eastern Finland, Kuopio, Finland

**Keywords:** Advanced practice nurse, Advanced practice nursing, Competence, Exploratory factor analysis, Nurse consultant, Nursing education

## Abstract

**Background:**

Determining the core competence of advanced practice nurses is foundational for promoting optimal design and implementation of advanced practice nursing roles. Core competencies specific to the contexts of the advanced practice nurse in Hong Kong have been developed, but not yet validated. Thus, this study aims to assess the construct validity of advanced practice nurse core competence scale in Hong Kong.

**Methods:**

We performed a cross-sectional study using an online self-report survey. Exploratory factor analysis was used to examine the factor structure of a 54-item advanced practice nurse core competence scale through principal axis factoring with direct oblique oblimin rotation. A parallel analysis was conducted to determine the number of factors to be extracted. The Cronbach’s α was computed to evaluate the internal consistency of the confirmed scale. The STROBE checklist was used as reporting guideline.

**Results:**

A total of 192 advanced practice nurse responses were obtained. Exploratory factor analysis led to the final 51-item scale with a three-factor structure, which accounted for 69.27% of the total variance. The factor loadings of all items ranged from 0.412 to 0.917. The Cronbach’s alpha of the total scale and three factors ranged from 0.945 to 0.980, indicating robust internal consistency.

**Conclusion:**

This study identified a three-factor structure of the advanced practice nurse core competency scale: client-related competencies, advanced leadership competencies, and professional development and system-related competencies. Future studies are recommended to validate the core competence content and construct in different contexts. Moreover, the validated scale could provide a cornerstone framework for advanced practice nursing roles development, education, and practice, and inform future competency research nationally and internationally.

**Supplementary Information:**

The online version contains supplementary material available at 10.1186/s12912-023-01203-1.

## Background

The rising demand for healthcare services and the shortage of health professionals, especially during the COVID-19 pandemic, are putting tremendous pressure on healthcare systems [1, 2]. Therefore, it is crucial to utilize manpower efficiently to guarantee the availability of high-quality and cost-effective healthcare services. As the largest occupational group in the health sector, nursing accounts for approximately 59% of health occupations [1]. One of the effective approaches is to make greater use of nurses in advanced practice [3, 4]. Advanced practice is a contemporary term emphasizing the progressive movement of graduate education within nursing [5]. The minimum entry requirement to advanced practice nursing roles is a master’s degree, helping advanced practice nurses (APNs) in more advanced practice activities including, education, management, leadership roles, policy, and research [6]. It is well documented that APNs have contributed significantly to improving patient care and outcomes and reducing healthcare costs [7, 8]. Implicated by the positive relationship between competence and performance [9, 10], clarifying and improving the core competencies of APNs could fundamentally optimize the implementation and impact of their roles. The APN core competencies in the Hong Kong context were developed by the Hong Kong Academy of Nursing (HKAN) [11]. However, to the best of our knowledge, they have not been validated to date. Internationally, achieving greater consistency among advanced practice nursing role competencies across countries is identified as an essential strategy for supporting the global development, effectiveness and impact, and geographic mobility of these roles [12]. Establishing a validated set of core APN competencies that are relevant to the healthcare contexts of Hong Kong is essential for the future development of the role in this region and for assessing competency alignment with the global advanced practice nursing community. Thus, this study set out to determine the construct validity of the proposed APN core competence scale.

The International Council of Nurses (ICN) [3] defines the APN as a registered nurse with at least a master’s degree who has acquired the expert knowledge base, advanced decision-making skills, and clinical competencies for expanded practice. Internationally, the two most common types of APNs are clinical nurse specialists (CNS) and nurse practitioners (NP) [3, 13]; however, it is recognized that advanced practice nursing role titles and competencies are shaped by the healthcare policies and contexts of the country in which the roles are established [14]. For example, nurse consultant (NC), who provides consultation services to improve nursing and other health care programs and standards, is also a title signifying the APN role that has been introduced in Hong Kong, Australia, and the UK [15, 16].

The APN in management is one of the 16 specialties in the Hong Kong advanced nursing practice. APNs in management and NCs share common capabilities HKAN developed in 2018 [8]. These competencies are well in line with international standards for APNs and facilitate greater role clarity and integration in Hong Kong [16]. Competencies are the attributes involving knowledge, skills, judgment, and attitudes that enable APNs to conduct a range of nursing tasks at an advanced professional level [17–19]. Establishing core competencies is foundational to defining and facilitating advanced practice nursing roles’ optimal development and performance [20]. Core competencies provide the basis for entry-level practice standards that are used to develop advanced practice nursing education programs and to set benchmarks for student evaluation, credentialing, and ongoing competency assessment [20, 21]. However, the development of APN competencies is widely heterogeneous, without a common practice [20].

Given the various dimensions of APN practice, the core competencies of APNs differ between organizations. The National Association of Clinical Nurse Specialists (NACNS) in the United States (US) began its efforts in 1995 to clarify the core competencies of CNSs and identified them in a statement issued in 1998 [22, 23]. The European Specialist Nurse Organisation (ESNO) identified competencies of the CNSs in 2015, including clinical role, patient relationship, patient teaching/coaching, mentoring, research, organization and management, communication and teamwork, ethic and decision making, leadership/policymaking, and public health [24]. In 2019, the most recent updated core competencies for CNSs were published by NACNS, which involves three domains: patient/client, nurses and nursing practice, and organizations and systems [23]. Recently, Finland also began its efforts in developing CNS core competency from four aspects: patient, clinical nursing leadership, organizational, and scholarship competencies [7]. Furthermore, Sastre-Fullana et al., [25] developed an APN competency assessment instrument in the Spanish context. The core competencies for NP were introduced by the National Organization of Nurse Practitioner Faculties (NONPF) in 1990 and updated in 2022, including nine competency areas: scientific foundation, leadership, quality, practice inquiry, technology and information literacy, policy, health delivery system, ethics, and independent practice [13]. The Canadian Nurse Association (CNA) identified the APN competencies in the first edition of Advanced Nursing Practice in 2000, which were divided into six categories: direct comprehensive care, health system optimization, education, research, leadership, and consultation and collaboration [26]. In the United Kingdom, the seven domains of competencies for NP were identified by the Royal College of Nursing (RCN) in 2002 based on those issued by NONPF, including management of patient health/illness status, the nurse-patient relationship, teaching-coaching function, professional role, managing and negotiating health care delivery systems, monitoring and ensuring the quality of health care practice, and cultural competence [27].

Overall, APN competency development is mainly concentrated in western countries, with limited description in Asian countries. In Singapore, the core competencies of APNs were organized into four domains, involving professional, legal and ethical nursing practice, management of care, leadership and management, and professional development [19]. The HKAN in Hong Kong described seven core domains of a competency framework for APNs, including managing clients with complex health conditions, enhancing therapeutic nurse-client relationships, demonstrating effective leadership and teamwork, enhancing quality assurance and improvement, managing and negotiating innovative and effective approaches to care delivery, enhancing professional attributes of general and advanced practice, and enhancing personal attributes [11].

Efforts have been spent on the development of core competencies for APNs worldwide. However, there are few validated APN core competencies within and across countries that are crucial to quantitatively assess the performance of APNs, guide the design of APN education curricula, and inform the policy. Existing valid APN competencies are restricted to a particular context like Canada [26], the US [28], Spain [25], and Finland [7]. Thus, this study was performed to evaluate an APN core competence scale (APN-CCS) in the Hong Kong context.

## Methods

### Aim

The objective of our study was to evaluate the construct validity of the APN-CCS by performing an exploratory fact analysis (EFA).

## Study Design

We performed a cross-sectional study using an online self-report survey, with data extracted from a large study comparing role activities amongst advanced practice nursing roles in Hong Kong. A 54-item self-reported APN-CCS was trialled using EFA [29]. This study was reported following the Strengthening the Reporting of Observation studies in Epidemiology (STROBE) checklist.

## Participants and data collection

Although Master’s degree is a minimum requirement for APN recognition [30], some APNs may not meet this standard due to lack of formal credentialing processes and rather recent development of the educational standards of the APN in Hong Kong [8]. Therefore, to obtain an adequate and representative sample, the potential APN participants in study were those who (1) were working in a clinical role and (2) were registered nurses with a diploma or baccalaureate degree in nursing and certified in a professional practice area, or APNs with a master’s or doctoral degree. Having obtained ethics approval, an invitation to engage in the study along with study details and the online questionnaire link were sent to advanced practice nursing postgraduate students (part-time students, who were all in advanced practice role but continuing to a higher college to gain specific knowledge to advance in management policies) (n = 313) at a local university and members of HKAN (n = 2546). The aim of the inclusion of both APN students and the members of HKAN was to coverage various competencies of advanced practice nursing role. Participant recruitment and data collection occurred between mid-November and March 2021. A total of 192 APNs completed the questionnaire. Supported by previous studies, this sample size was adequate to execute factor analysis [31].

## Study instrument

The original 54-item HKAN Competence Statements for Advanced Practice Nurses was used as APN-CCS [11]. The statements delineated seven core domains of APNs’ competence framework for: *Managing clients with complex health conditions* (11 items); *Enhancing therapeutic nurse-client relationship* (8 items); *Demonstrating effective leadership and team work* (6 items); *Enhancing quality assurance and improvement* (6 items); *Managing and negotiating innovative and effective approaches to care delivery* (7 items); *Enhancing professional attributes of general and advanced practice* (14 items); *Enhancing personal attributes* (2 items). The HKAN Competence Statement was originally developed based on Hong Kong and international literature [16]. In Hong Kong, the competencies of the nurse specialists in the 14 areas in alignment of the HKAN Colleges were reviewed. The international literature included those from Australia, United States and the United Kingdom. The local and international competences were compared and contrasted in the development. An expert panel composes of nurse specialists, nurse managers, and nurse academics met multiple times, discussed and deliberated to derive the set of competencies to reflect the actual practice locally and the standards internationally. The 54-item HKAN Competency Statement were validated by a panel of four experts to confirm if the items in the questionnaires were valid and relevant to the practice of APNs in the context where they practice. All items had an item content validity index (I-CVI) of 1 and the scale content validity index (SCVI) was also 1, which indicated total agreement. The APN-CCS was employed to measure the use of the APN competence in a typical month with a five-point, Likert-style scale (0 = Not at all, 1 = Little extent, 2 = Some extent, 3 = Great extent, 4 = very great extent). A higher score indicates greater extent of usage on the related competency. The Cronbach’s α of this 54-item scale was 0.811 in our study.

### Data analysis

Statistical analysis was performed using IBM SPSS 25.0 for data entry and statistical analysis. There was no missing data. The demographic characteristics of the participants were described using descriptive statistics by means and standard deviations as well as frequencies and percentages.

The factor structure of APN core competence as measured by APN-CCS was examined by EFA through principle axis factoring (PAF). Since factors cannot be presumed to be independent in such type of data, direct oblique oblimin rotation was applied to rotate the EFA factors [31]. Sampling adequacy was evaluated by the Kaiser-Meyer-Olkin (KMO) index and Bartlett’s test of sphericity. The cut-off value for the KMO statistic was ≥ 0.8, and for the cut-off value, Bartlett’s test of sphericity was < 0.05, indicating that the sample was adequate and suitable for performing factor analysis [32]. To identify the number of factors to be extracted, parallel analysis was conducted [33, 34], by applying the SPSS syntax [35], with 1000 repetitions. The number of factors to be extracted was also confirmed by conventional method, namely, scree plot. Items with factor loadings less than 0.4 on all the extracted factors were removed sequentially, and such a threshold represents the smallest meaningful correlation between the item and factor [32]. Furthermore, items with cross-loading over 0.4 on more than one factor and a ratio of loading higher than 75% were also removed sequentially [36, 37 ]. The Cronbach’s α was calculated to evaluate the internal consistency of the confirmed scale. The Cronbach’s α coefficient of 0.7 or above is considered acceptable, and a value ≥ 0.8 are preferable [38, 39].

## Ethical considerations

The University of Eastern Finland Committee on Research Ethics granted ethical approval of the study (statement number 22/2018), and The HKAN approved the conduction of the study through its membership platform. Data were kept strictly confidential and processed following the policies of the HKAN. No personal or identifying data were collected. The study was conducted under the principles described in the Declaration of Helsinki. Study information was provided, and informed consent was obtained before participants completed the questionnaire.

## Results

### Sample characteristics

Totally, 192 APNs returned the self-reported survey. The response rate was 7%. Table 1 presents a summary of the demographic characteristics of the APN participants. The majority of APN participants were women over the age of 46 years. They also had substantive work experience in nursing and had been in their current position for over six years. Most of the participants had also obtained their master’s certificates and were working in an acute care hospital. Among the 192 APN respondents, 14 position titles were reported, such as advanced practice midwife, nursing officer, ward manager, and NC. These position titles were then classified into three role categories of APN, APN in management, and NC to align the two streams of career progression (management or clinical) and to accommodate the second (APNs/nurse managers) and third (NCs) tiers of the nursing professional career ladder [40].

**Table 1:** Characteristics of the APN participants (n = 192)

Characteristics	Mean ± SD or n (%)
Age (years)	46.83 ± 8.00
Female	155 (80.7)
Education level	
Bachelor’s degree	14 (7.3)
Master’s degree	172 (89.6)
Doctoral	6 (3.1)
Hospital type	
Acute	154 (80.2)
Rehabilitation	19 (9.9)
Day care	6 (3.1)
Not applicable	11 (5.7)
Current position title	
Advanced practice nurse	106 (55.2)
Advanced practice nurse in	52 (27.1)
Management	
Nursing consultant	34 (17.7)
Years in nursing	25.59 ± 7.97
Years in current position	6.22 ± 4.90

Note: data are presented in mean and standard deviation (SD) or frequency (n) and percentage (%)

## Exploratory factor analysis

The KMO index was 0.948, and the *p*-value of Bartlett’s test of sphericity was < 0.001, suggesting that data were suitable for EFA [32]. Table 2 shows the actual eigenvalues of the study sample and the mean and 95th percentile of the generated eigenvalues from the parallel analysis. The results show that only the first three actual eigenvalues were larger than both the mean and 95th percentile of those generated by the parallel analysis. Therefore, the parallel analysis suggested that three factors could be extracted. However, the scree plot suggested a four-domain structure. Both three and four factors were thus tried to decide the finial structure. Fixing 3 or 4 factors to be extracted at the beginning of the data analysis finally led a three-factor structure of APN-CCS. This may due to the rule that items with factor loadings less than 0.4 on all the extracted factors were removed.

**Table 2:** Actual eigenvalues of the study sample and mean and 95th percentile of generated eigenvalues from a parallel analysis with 1000 repetitions

Eigenvalues		Actual	Mean (generated)	95th percentile (generated)
1		25.886	2.173	2.294
2		7.379	2.048	2.142
3		2.063	1.958	2.033
4		1.557	1.879	1.949
5		1.138	1.808	1.870
6		1.122	1.744	1.803
7		0.947	1.684	1.736
8		0.812	1.627	1.678
9		0.672	1.574	1.622
10		0.639	1.525	1.571

Only the first ten eigenvalues are shown

Three (items 29,39,53) out of 54 items of APN-CCS with loading values under 0.40 were deleted sequentially after the initial analysis. These three items were: “*Manages complaints and monitors malpractice*”, “*Applies principles of epidemiology and demography in clinical practice*”, and “*Analyzes situation critically and draws relationship among issues.*” Thereafter the PAF was re-conducted, which led to the final 51-item APN-CCS, without any cross-loading items. The final 51-item APN-CCS with a three-factor structure accounted for 69.27% of the total variance. The factor loadings of all items ranged from 0.412 to 0.917 (Table 3).

**Table 3:** Principle axis factoring analysis (Oblimin with Kaiser Normalization) of the APN-CCS

APN-CCS items	1	2	3
**Domain 1: Client-related competencies (19-item)**			
1. Manages complete episode of care for complicated health cases and refers aspects of care to own and other professions.	0.781		
2. Provides case management services to meet multiple client health care needs.	0.809		
3. Plans and implements diagnostic strategies and therapeutic interventions to help clients with unstable and complex health care problems regain stability and restore health in collaboration with the client and multidisciplinary health care team	0.820		
4. Rapidly assesses client’s unstable and complex health care problems through synthesis and prioritization of historically and immediately derived data.	0.905		
5. Selects, may perform, and interprets common screening and diagnostic laboratory tests.	0.856		
6. Diagnoses and manages acute and chronic diseases while attending to the illness experience.	0.871		
7. Diagnoses unstable and complex health care problems utilizing collaboration and consultation with the multidisciplinary health care team as indicated by setting, specialty, and individual knowledge and experience.	0.832		
8. Reviews medication regime and counsels clients concerning drug regimens, drug side effects, and interactions.	0.838		
9. Assesses and adjusts plans for continuous management of client’s health status by monitoring variation in wellness and illness.	0.848		
10. Obtains specialist and referral care for clients while remaining the primary care provider.	0.823		
11. Monitors client data base for follow-up, consultation, referral, and outcomes.	0.825		
12. Demonstrates skills in promoting therapeutic interaction to effect clients’ change in health behavior.	0.892		
13. Provides guidance and counseling regarding symptom management.	0.857		
14. Provides emotional and informational support to clients and their families.	0.870		
15. Uses human skills to enhance effectiveness of relationship.	0.767		
16. Applies principles of self-efficacy/empowerment in promoting behavior change.	0.679		
17. Monitors and reflects own emotional response to client interaction and uses as data to further therapeutic interaction.	0.796		
18. Facilitates staff to debrief on overwhelming emotion and grief associated with nurse-client relationship.	0.615		
19. Communicates a sense of “being present” with the client.	0.759		
**Domain 2: Advanced leadership competencies (11 items)**			
20. Coordinates human and environmental resources necessary to manage rapidly changing situations.		0.543	
22. Empowers staff to assume increasing responsibilities for complicated client care with delegation, support and supervision.		0.555	
23. Provides leadership in the interdisciplinary team through the development of collaborative practices or innovative partnerships		0.686	
24. Demonstrate effective leadership skills and be able to exert influence in a group.		0.819	
25. Provides leadership in professional activities.		0.718	
40. Promotes and fosters ethical practice and advocacy for clients.		0.430	
46. Demonstrates expertise on area(s) of nursing. Be a resource person for referrals in these areas.		0.444	
47. Interprets own professional strengths, role, and scope of ability to peers, clients and colleagues.		0.666	
48. Acts as a role model and sets exemplary standard of professional behaviors.		0.917	
49. Supports socialization, education, and training of novice practitioners by serving as a preceptor, role model and mentor.		0.684	
50. Motivates and support staff to be self-developing and achieve higher professional goals.		0.786	
**Domain 3: Professional development and system-related competencies (21 items)**			
21. Leads hospital/community health education and promotional activities.			0.692
26. Leads the on-going process of setting and revising guidelines, protocols, standards and contingency plan.			0.619
27. Develops a tracking system within the practice to ensure that clients receive appropriate preventive services.			0.705
28. Monitors peers, self and delivery system through Quality Assurance, Total Quality Management, as part of Continuous Quality Improvement			0.567
30. Benchmarks various care programs with outcome measures and advise on clinical management or recommend review of intervention as indicated			0.825
31. Initiates and implements quality improvement strategies and clinical audits in collaboration with various health disciplines.			0.854
32. Employs appropriate diagnostic and therapeutic interventions and regimens for specific client groups with attention to safety, cost, acceptability, efficacy and cost-effectiveness.			0.673
33. Suggests implementation of evidenced-based practice and facilitates changes.			0.762
34. Uses evidence and rationale to leverage senior and other on decision making			0.774
35. Contributes to the development of overall client care delivery system and adopts appropriate nursing models in system to achieve optimal outcomes.			0.781
36. Re-engineers the work process.			0.685
37. Establishes detailed implementation schedules, resources planning, achievement indicators, and monitoring mechanism to support the service development plan.			0.849
38. Envisions change impacts. Be prepared to take reasonable risk to facilitate change and open to innovations			0.814
41. Applies/develops a theory-bases conceptual framework to guide practice			0.623
42. Attains self-advancement professionally through initiating and involving in evidence based practice and research activities.			0.734
43. Masters the application of advanced health care technology in specific area and shows knowledge on the evidence found.			0.582
44. Critically evaluates and applies research studies pertinent to client care management and outcomes			0.816
45. Applies/conducts research studies pertinent to primary care and/or specialty practice management.			0.915
51. Interprets and markets the advanced practicing nurse role to the public and other health care professionals.			0.412
52. Participates in legislative and policy-making activities which influence advanced nursing practice and health services.			0.730
54. Maintains active membership in professional organization.			0.514

The results of EFA suggested that two original domains of competencies related to: “*Managing clients with complex health conditions*” and “*Enhancing therapeutic nurse-client relationship*” could be combined. The original nineteen items were loaded to this factor, with factor loading ranging from 0.615 to 0.905. Based on the contents, this factor was renamed as “*client-related competencies*” to account for the items in the factor.

Five items from the original domain of “*Demonstrating effective leadership and team work*”, together with six from “*Enhancing professional attributes of general and advanced practice*” were combined into the second factor. Based on the contents, this 11-item factor was renamed as “*advanced leadership competencies*” to tie these 11 items better. The factor loading ranged from 0.430 to 917.

For the last factor, the results of EFA suggested the combination of four original domains of competencies: “*Enhancing quality assurance and improvement*”, “*Managing and negotiating innovative and effective approaches to care delivery*”, “*Enhancing professional attributes of general and advanced practice*”, and “*Enhancing personal attributes*”. Twenty-one items were loaded to this factor, with factor loading ranging from 0.412 to 0.915. Based on the contents, this factor was renamed as “*professional development and system-related competencies*” to illustrate the items in the factor.

The Cronbach’s alpha of the 51-item APN-CCS was 0.980, indicating a robust internal consistency. For the three factors (sub-scale), factor 1 showed the highest value of 0.976, followed by factors 3 (α = 0.970) and factor 2 (α = 0.945).

## Discussion

This study aimed to establish a validated effective quantitative instrument targeted at evaluating the core competencies of APNs in Hong Kong. Study results led to a three-factor structure of the 51-item APN-CCS. The original seven domains of APN competency [11] were condensed down to three factors and proposed competency domains, including (1) client-related competencies, (2) advanced leadership competencies, and (3) professional development and system-related competencies.

The construct validity of the APN-CCS scale was evaluated by performing the EFA, which reduces variable complexity to greater simplicity [41]. Based on the EFA results, the total variance explained by the proposed three-factor structure was within the recommended range for multi-dimensional measurement[42], indicating the APN-CCS provides adequate coverage in evaluating APN core competencies.

There is a similarity between the proposed three-factor structure and APN competency domains identified in Finland, which include patient, clinical nursing leadership, organizational, and scholarship competencies [7]. Similarly, the organizing framework for CNS core competencies in the US describes CNS practice as integrated across three spheres: patient/client, nurse and nursing practice, and the organizational/system [23]. Consistent with these categories, Domain 1 (client-related competencies) of the APN-CCS represent patient impacts, Domain 2 (advanced leadership competencies) signifies leadership impacts, and Domain 3 (professional development and system-related competencies) indicates professional and organization/system impacts [16].

The client-related domain reinforces the long-standing view of clinical practice as the foundation for advanced practice nursing roles and the APN as a clinical expert [7, 15, 23, 43]. In domain, competencies related to “*provides case management services to meet multiple client health care needs*” (item 2), “*monitors client data base for follow-up, consultation, referral and outcomes*” (item 11), and “*communicates a sense of ‘being present’ with the client*” (item 19), reflect enhanced APN competencies required to assess, manage, and coordinate care to accommodate client complex health care needs, promote client self-care, and to establish effective partnerships with clients [7].

Given their enhanced clinical knowledge, skills, and expertise, APNs are ideally positioned to provide nursing leadership roles [44]. Thus, it is not surprising that leadership is consistently recognized as a core domain within the APN competency framework [20]. Domain 2 of APN-CCS is related to APN competencies in demonstrating effective leadership and teamwork. This domain also contains competencies like acting as a model and mentor to others and serving as a consultant or resource person for patients, nurses, and other health professionals. These competencies are mirrored in items such as “*act as a role model and sets exemplary standard of professional behaviours*” (item 48), “*demonstrates expertise on area(s) of nursing, and be a resource person for referrals in these areas*” (item 46). Serving as a consultant resource person, APNs can provide specific expert clinical advice, facilitate problem solving for complex clinical issues, and consult on professional and organizational development.

Domain 3 of the proposed APN-CCS represents APN core competencies at the professional and organizational level. Compared with general and specialist nurses, APNs are more involved in non-clinical role activities related to nursing professional development, research, and publication [7]. Previous studies have also identified that involvement in research and research-related activities differentiates APNs from nurses in non-advanced roles [45, 46]. Research-related competencies enable APNs to enhance patient care through the use of scientific evidence [47]. These activities are consistent with Domain 3 items, such as “*critically evaluates and applies research studies pertinent to client care management and outcomes*” (item 44). Additionally, the range of Domain 3 covers far beyond research to include organizational and system competencies, such as leading quality improvement initiatives, promoting the uptake of innovative and effective practices, and assessing the cost-effectiveness of care. The organizational/system impacts have also been regarded as one of the focus areas of the advanced practice nursing role [7, 48]. These competencies reflect APNs’ competence in clarifying the value of nursing at the organizational/system level and influencing decision-making at the system level to improve patient outcomes. Investing in evidence-based practice and strategic development is also an important part of the APNs’ role [15, 23]. For example, the items like “*suggests implementation of evidenced-based practice and facilitates changes*” (item 33) and “*uses evidence and rationale to leverage senior and other in decision making*” (item 34) are included in this domain. The activities of APNs at the system level contribute to the optimal operation of the nursing service, including role advocacy, enhancing innovative patient care, and promoting optimal patients progress through the healthcare system [47].

Having a higher level of education among APNs allows them to spend more time in advanced practice activities. According to Wilkinson et al., [6]educational standards are necessary to improve the consistency of advanced nursing roles across countries. Therefore, it is worth noting that efforts have been made worldwide to describe the APN core competencies. Despite this, there are still few rigorously validated APN core competencies standards. A valid and reliable APN core competencies tool was developed in the Spanish context [25]. Rigorous validation processes were conducted in the US and Finland to test the core competencies for the specific APN role, CNS [7, 28]. This study in the Hong Kong context would be thus valuable in reinforcing the international evidence base for global APN competency development and implementation and may introduce new directions for shaping the role of APNs. Furthermore, policymakers and scholars could consider use these validated APN core competencies to compare APN services in different contexts, nationally or internationally, and analyse their links to the outcomes of the services provided.

The validated 51-item APN-CCS would be used to measure the usage of the APN competence in a typical month with a five-point, Likert-style scale (0 = Not at all, 1 = Little extent, 2 = Some extent, 3 = Great extent, 4 = very great extent), yielding a potential range of total scores from 0 to 204. The client-related domain consisted of 19 competency items. Thus, the potential range for usage scores would be from 0 to 76. The second domain of advanced leadership competencies consisted of 11 items; thus, the potential score would range from 0 to 44. The professional and organizational domain consisted of 21 competency items. Therefore, the potential range for scores would be from 0 to 84. A higher score indicates greater extent of usage on the related competency. The validated tool could provide healthcare organizations with a cornerstone framework for competency assessment with the potential application: (1) in clinical practice and management organizational levels to comprehensively guide and assess APN performances; (2) in advanced practice nursing education programs to promote optimal development and to evaluate students’ performance; (3) in health policies for advanced practice nursing roles description and implementation; and (4) in APN research area to inform future competency research nationally and internationally.

This study had some limitations. First, the sample of this study was recruited from APN students and the members of HKAN. This method may limit the generalization of the study results to all APNs in Hong Kong. Moreover, 7.3% of the nurses who participated in this study had not completed a master’s degree as an expected requirement for advanced practice. It is possible that some of these nurses were APN graduate students who were already working in an advanced role. The minimum APN educational standard of master’s degree appear to be agreed upon by the APN community, however, issues with meeting this qualification remain, with studies reporting variability in APN education [46, 49]. Completion of a master’s degree continues to be important for promoting the optimal implementation of APN role domains [3, 50, 51]. Secondly, an online questionnaire was applied in this study, and the response rate was low despite multiple reminders, which may also be due to the practice requirements of nurses in the global COVID-19 pandemic. Thus, further efforts to validate the APN-CCS in a broader sample of APNs in Hong Kong may be warranted. The study offers suggestions on the developing of APN-CCS strictly based on EFA results, as the following steps, further validity and reliability testing, such as confirmatory factor analysis, known-group validity (construct), and test-retest reliability, should be conducted to further strengthen the psychometric properties of APN-CCS and to possibly build a more parsimonious APN core competencies framework.

## Conclusion

Core APN competencies proved an essential framework for advanced practice nursing role development, education, and practice. A competency validation survey provided a valid method to measure APN competence reliably and help to define practice standards and possibly contribute towards consistency of the APN practice. This study provides the first evaluation of the construct validity of APN-CCS in Hong Kong. Our results identified a three-factor structure of APN-CCS: client-related competencies, advanced leadership competencies, and professional development and system-related competencies. Future studies should validate the core competence content and construct in a different context.

## Electronic supplementary material

Below is the link to the electronic supplementary material.

Supplementary Material 1

## Data Availability

The data that support the findings of this study are available from the corresponding author upon reasonable request.

## Abbreviations

APNs: Advanced Practice Nurses
APN-CCS: APN Core Competence Scale
CNA: Canadian Nurse Association
CNS: Clinical Nurse Specialists
EFA: Exploratory Fact Analysis
ESNO: European Specialist Nurse Organisation
HKAN: Hong Kong Academy of Nursing
KMO: Kaiser-Meyer-Olkin
ICN: International Council of Nurses
NACNS: National Association of Clinical Nurse Specialists
NC: Nurse Consultant
NONPF: National Organization of Nurse Practitioner Faculties
NP: Nurse Practitioners
PAF: Principle Axis Factoring
RCN: Royal College of Nursing
STROBE: Strengthening the Reporting of Observation studies in Epidemiology
US: United States
WHO: World Health Organization.
