# Supplementary material for: Construct validity of advanced practice nurse core competence scale: an exploratory factor analysis
Source: BMC Nurs. 2023 Mar 2;22:57. doi: 10.1186/s12912-023-01203-1 (PMC9979114; doi:10.1186/s12912-023-01203-1)
Supplement: Supplementary file 1 — Supplementary Material 1 [file 12912_2023_1203_MOESM1_ESM.docx]

**Appendix.** The original version of 54-item APN-CCS

For each of the following clinical care competencies, please indicate the extent of time in your current position, that you spend on each one during a typical month. The scale is as follows:

4=to a very great extent

3=to a great extent

2=to some extent

1=to a little extent

0=not at all

Please choose the appropriate response for each item

| **APN-CCS (Items)** |  |  |  |  |  |
| --- | --- | --- | --- | --- | --- |
| **Managing clients with complex health conditions (11 items)** | Very great extent | Great extent | Some extent | Little extent | Not at all |
| 1. Manages complete episode of care for complicated health cases and refers aspects of care to own and other professions. |  |  |  |  |  |
| 1. Provides case management services to meet multiple client health care needs. |  |  |  |  |  |
| 1. Plans and implements diagnostic strategies and therapeutic interventions to help clients with unstable and complex health care problems regain stability and restore health in collaboration with the client and multidisciplinary health care team |  |  |  |  |  |
| 1. Rapidly assesses client’s unstable and complex health care problems through synthesis and prioritization of historically and immediately derived data. |  |  |  |  |  |
| 1. Selects, may perform, and interprets common screening and diagnostic laboratory tests. |  |  |  |  |  |
| 1. Diagnoses and manages acute and chronic diseases while attending to the illness experience. |  |  |  |  |  |
| 1. Diagnoses unstable and complex health care problems utilizing collaboration and consultation with the multidisciplinary health care team as indicated by setting, specialty, and individual knowledge and experience. |  |  |  |  |  |
| 1. Reviews medication regime and counsels clients concerning drug regimens, drug side effects, and interactions. |  |  |  |  |  |
| 1. Assesses and adjusts plans for continuous management of client’s health status by monitoring variation in wellness and illness. |  |  |  |  |  |
| 1. Obtains specialist and referral care for clients while remaining the primary care provider. |  |  |  |  |  |
| 1. Monitors client data base for follow-up, consultation, referral, and outcomes. |  |  |  |  |  |
| **Enhancing therapeutic nurse-client relationship (8 items)** | Very great extent | Great extent | Some extent | Little extent | Not at all |
| 1. Demonstrates skills in promoting therapeutic interaction to effect clients’ change in health behavior. |  |  |  |  |  |
| 1. Provides guidance and counseling regarding symptom management. |  |  |  |  |  |
| 1. Provides emotional and informational support to clients and their families. |  |  |  |  |  |
| 1. Uses human skills to enhance effectiveness of relationship. |  |  |  |  |  |
| 1. Applies principles of self-efficacy/empowerment in promoting behavior change. |  |  |  |  |  |
| 1. Monitors and reflects own emotional response to client interaction and uses as data to further therapeutic interaction. |  |  |  |  |  |
| 1. Facilitates staff to debrief on overwhelming emotion and grief associated with nurse-client relationship. |  |  |  |  |  |
| 1. Communicates a sense of “being present” with the client. |  |  |  |  |  |
| **Demonstrating effective leadership and team work (6 items)** | Very great extent | Great extent | Some extent | Little extent | Not at all |
| 1. Coordinates human and environmental resources necessary to manage rapidly changing situations |  |  |  |  |  |
| 1. Leads hospital/community health education and promotional activities. |  |  |  |  |  |
| 1. Empowers staff to assume increasing responsibilities for complicated client care with delegation, support and supervision. |  |  |  |  |  |
| 1. Provides leadership in the interdisciplinary team through the development of collaborative practices or innovative partnerships |  |  |  |  |  |
| 1. Demonstrate effective leadership skills and be able to exert influence in a group. |  |  |  |  |  |
| 1. Provides leadership in professional activities. |  |  |  |  |  |
| **Enhancing quality assurance and improvement (6 items)** | Very great extent | Great extent | Some extent | Little extent | Not at all |
| 1. Leads the on-going process of setting and revising guidelines, protocols, standards and contingency plan |  |  |  |  |  |
| 1. Develops a tracking system within the practice to ensure that clients receive appropriate preventive services. |  |  |  |  |  |
| 1. Monitors peers, self and delivery system through Quality Assurance, Total Quality Management, as part of Continuous Quality Improvement |  |  |  |  |  |
| 1. Manages complaints and monitors malpractice |  |  |  |  |  |
| 1. Benchmarks various care programs with outcome measures and advise on clinical management or recommend review of intervention as indicated. |  |  |  |  |  |
| 1. Initiates and implements quality improvement strategies and clinical audits in collaboration with various health disciplines |  |  |  |  |  |
| **Managing and negotiating innovative and effective approaches to care delivery (7 items)** | Very great extent | Great extent | Some extent | Little extent | Not at all |
| 1. Employs appropriate diagnostic and therapeutic interventions and regimens for specific client groups with attention to safety, cost, acceptability, efficacy and cost-effectiveness. |  |  |  |  |  |
| 1. Suggests implementation of evidenced-based practice and facilitates changes. |  |  |  |  |  |
| 1. Uses evidence and rationale to leverage senior and other on decision making |  |  |  |  |  |
| 1. Contributes to the development of overall client care delivery system and adopts appropriate nursing models in system to achieve optimal outcomes. |  |  |  |  |  |
| 1. Re-engineers the work process. |  |  |  |  |  |
| 1. Establishes detailed implementation schedules, resources planning, achievement indicators, and monitoring mechanism to support the service development plan. |  |  |  |  |  |
| 1. Envisions change impacts. Be prepared to take reasonable risk to facilitate change and open to innovations |  |  |  |  |  |
| **Enhancing professional attributes of general and advanced practice (14 items)** | Very great extent | Great extent | Some extent | Little extent | Not at all |
| 1. Applies principles of epidemiology and demography in clinical practice. |  |  |  |  |  |
| 1. Promotes and fosters ethical practice and advocacy for clients. |  |  |  |  |  |
| 1. Applies/develops a theory-bases conceptual framework to guide practice |  |  |  |  |  |
| 1. Attains self-advancement professionally through initiating and involving in evidence based practice and research activities. |  |  |  |  |  |
| 1. Masters the application of advanced health care technology in specific area and shows knowledge on the evidence found. |  |  |  |  |  |
| 1. Critically evaluates and applies research studies pertinent to client care management and outcomes |  |  |  |  |  |
| 1. Applies/conducts research studies pertinent to primary care and/or specialty practice management. |  |  |  |  |  |
| 1. Demonstrates expertise on area(s) of nursing. Be a resource person for referrals in these areas. |  |  |  |  |  |
| 1. Interprets own professional strengths, role, and scope of ability to peers, clients and colleagues. |  |  |  |  |  |
| 1. Acts as a role model and sets exemplary standard of professional behaviors. |  |  |  |  |  |
| 1. Supports socialization, education, and training of novice practitioners by serving as a preceptor, role model and mentor. |  |  |  |  |  |
| 1. Motivates and support staff to be self-developing and achieve higher professional goals. |  |  |  |  |  |
| 1. Interprets and markets the advanced practicing nurse role to the public and other health care professionals. |  |  |  |  |  |
| 1. Participates in legislative and policy-making activities which influence advanced nursing practice and health services. |  |  |  |  |  |
| **Enhancing personal attributes (2 items)** | Very great extent | Great extent | Some extent | Little extent | Not at all |
| 1. Analyzes situation critically and draws relationship among issues. |  |  |  |  |  |
| 1. Maintains active membership in professional organization. |  |  |  |  |  |
